# The diagnosis and treatment of tibial intercondylar chondroblastoma

**DOI:** 10.6061/clinics/2018/e540

**Published:** 2018-11-16

**Authors:** Zhengming Yang, Huimin Tao, Zhaoming Ye, Xin Huang, Nong Lin, Minfei Yang

**Affiliations:** I Orthopedics Department, Second Affiliated Hospital of the Zhejiang University School of Medicine, Hangzhou, Zhejiang, 310090; II Emergency Department, Second Affiliated Hospital of the Zhejiang University School of Medicine, Hangzhou, Zhejiang, 310090

**Keywords:** Tibial Intercondylar Region, Chondroblastoma, Diagnosis, Treatment

## Abstract

**OBJECTIVES:**

To investigate the diagnosis and treatment of tibial intercondylar chondroblastoma.

**METHODS:**

We retrospectively analyzed the diagnosis and treatment of 12 patients with tibial intercondylar chondroblastoma admitted to the orthopedics department from May 2011 to February 2016; among them were 4 males and 3 females aged 10-19 years, with an average age of 15.7 years. Tibial intercondylar chondroblastoma was on the left and right side in 7 and 5 cases, respectively. The preoperative average Lysholm score of the knee joint was 68 (42-87). A posteromedial approach was applied in all cases. The incisions were approximately 5-8 cm in length. Complete curettage and inactivation were performed after fenestration, and allogeneic bone grafts were transplanted. Then, the posterior cruciate ligament insertion was fixed with 5.0 suture anchors. All patients were followed up with regularly to monitor for tumor recurrence, observe bone graft healing, and reassess the Lysholm score of the knee.

**RESULTS:**

Patients were followed for 7-55 months, and the median follow-up time was 19 months. One patient experienced tumor relapse 4 months after the operation. Incision, inactivation and cementation were performed. Then, the bone was fixed with anchors. In the other 11 patients, the bone graft healed over an average period of 6.2 months (4-10 months), with good functional recovery postoperatively. The average postoperative Lysholm score of the knee was 91 (81-95).

**CONCLUSION:**

Tibial intercondylar chondroblastoma has unique clinical and imaging characteristics and can effectively be treated by curettage followed by the inactivation, transplantation and fixation of allogeneic bone grafts with suture anchors through a posteromedial approach.

## INTRODUCTION

Chondroblastoma is a rare primary benign bone tumor, accounting for approximately 1% of primary benign bone tumors 1. Many benign and malignant bone tumors tend to arise in the periphery of the knee joint. However, as tumors in the tibial intercondylar region are rare, they are easily misdiagnosed or missed altogether because of the unique pathological location. The only effective treatment is surgical excision. In the treatment process, tumors must be completely removed to avoid relapse and joint dysfunction. Twelve patients with tibial intercondylar chondroblastoma were admitted to our orthopedics department from May 2011 to February 2016. The diagnosis and treatment of this disease are discussed below.

## MATERIALS AND METHODS

### Ethics statement

Ethical approval was granted by the medical ethics committee of The Second Affiliated Hospital of the Zhejiang University School of Medicine.

### General materials

Twelve patients with tibial intercondylar chondroblastoma were admitted to our orthopedics department from May 2011 to February 2016. Among them were 9 males and 3 females aged 10-19 years, with an average age of 15.7 years. Tibial intercondylar chondroblastoma was on the left and right sides in 7 and 5 cases, respectively. The course of these cases ranged from 6 months to 2 years . The main clinical characteristics were pain and swelling of the knee joint complicated with limited joint function. The preoperative average Lysholm score 2 of the knee joint was 68 (42-87).

After admission, the affected side of all patients was imaged by X-rays, computed tomography (CT) and magnetic resonance imaging (MRI). The epiphyseal plates of 10 patients were not closed. In 8 cases, the lesion was located inside the epiphysis, and in 4 cases, the lesion extended from the epiphysis to the metaphysis through the epiphyseal plate. Close to the articular cartilage, all lesions under the articular surface were below the posterior cruciate ligament insertion. All lesions were manifested by a round or oval osteolytic focus in which calcification could be observed. MRI showed an inflammatory reaction around the lesions, edema of the epiphysis and soft tissues, and joint effusion.

Nine patients with no preoperative biopsy were diagnosed with chondroblastoma by the clinical and imaging evaluations. CT-guided needle biopsy was performed in the other 3 early cases. Frozen pathological examination was conducted intraoperatively in 9 cases and confirmed the chondroblastoma. The postoperative pathological finding was chondroblastoma in all cases.

### Surgical procedures

A posteromedial approach was applied in all cases. The incisions were approximately 5-8 cm in length. The medial head of the gastrocnemius was pulled to the fibular side, and the articular capsule was then incised to expose the tibial intercondylar posterior cruciate ligament insertion. The surrounding tissues were covered with gauze. After the retractor was withdrawn, fenestration was performed below the exposed posterior cruciate ligament with a diameter of approximately 1.0-1.5 cm. Tumor tissues were curetted through the bone window, and the bone on the tumor cavity wall was removed by grinding in all directions. Phenol and alcohol were used repeatedly to wash the bone wall; 5.0 suture anchors were fixed but not to the epiphyseal plate. Allograft bone granules were implanted into the bone defects 3. In addition, the sutures were fixed on the posterior cruciate ligament insertion. Finally, the articular capsule was closed.

### Postoperative management

The patients could move the knee joint passively starting the second day after the operation and were asked to exercise slowly and gently. The patients began using crutches beginning the second week postoperatively and carrying loads one and a half months after the operation.

## RESULTS

**Figure 1:**
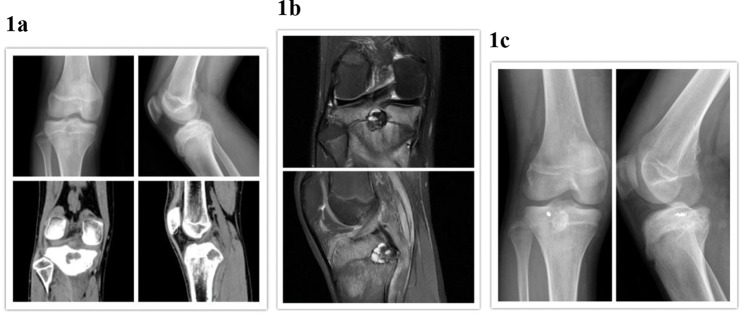
Male, 15, right knee pain for 3 months. **1a** Preoperative X-ray films and CT scans showing a round osteolytic focus below the right tibial intercondylar posterior cruciate ligament just below the circular osteolytic lesion. There was sclerosis at the boundary, and calcification was observed in the lesion. **1b** Preoperative MRI scans showing an inflammatory reaction around the lesion and edema of the surrounding bone marrow and soft tissues. **1c** X-ray films at four months after the operation suggesting that the grafted bone granules in the lesion had integrated with each other, resulting in an unclear boundary between the granules and the host bone.

The patients received reexaminations at one month after the operation, every 3 months after bone fusion, and once a year thereafter. X-ray examination of the knee joint (CT examination in some patients) was performed to monitor for tumor recurrence and observe bone graft healing. Bone graft healing was confirmed when no absorbing cavity was present in the bone graft area and the boundary between the grafted bone and the host bone was indistinct. The patients were followed for 7-55 months, and the median follow-up time was 19 months. One patient experienced tumor recurrence four months after the operation. Incision, inactivation and cementation were performed, and then the bone was fixed with anchors. Recurrence was not found in subsequent follow-up examinations. In the other 11 patients, the bone graft healed over an average of 6.2 months (4-10 months). Relative to before the operation, pain in the knee joint was relieved after the operation, and the function of the knee had recovered to normal. Myositis ossificans was not observed. No growth malformations or inequalities were found in the lower extremities during the postoperative follow-up visits. The postoperative average Lysholm score of the knee joint was 91 (81-95). Images from typical cases are shown in Figure 1.

## DISCUSSION

### Clinical characteristics

Stage 2-3 chondroblastomas are active and aggressive tumors, according to the Enneking system 4. Chondroblastoma in the epiphysis or bone ends often leads to severe inflammatory responses in the joints of the lesion, which manifest as joint pain and swelling. Patients with tibial intercondylar chondroblastoma all feel pain at first, followed by various degrees of swelling and limited movement of the knee joint. In severe cases, patients feel pain so intensely that the knee cannot be flexed and moderate painkillers are needed for relief. However, missed diagnosis and misdiagnosis are frequent in clinical practice due to the low incidence and unique pathological location of this condition; in such cases, patients are commonly diagnosed with synovitis.

The characteristics of tibial intercondylar chondroblastoma in this group of patients are summarized as follows. This disease often occurs among adolescents and is more common in males than females. It mainly manifests as pain in the knee joint complicated with limited movement. The imaging characteristics include a round or oval osteolytic focus with a clear boundary, and swelling can be observed on the side adjacent to the articular surface. Calcification can be found in the center of the lesion with no periosteal reaction or soft tissue mass. CT displays the thinness and penetration of cortical bone more accurately. Chondroblastoma shows a low- or medium-intensity signal on T1-weighted MRI and a significantly higher-intensity signal on T2-weighted MRI with corresponding low-intensity, calcified interior regions. MRI also shows significant inflammation around the joints, manifested by edema of the bone marrow and soft tissue. Joint effusion can be observed in some cases.

### Preoperative biopsy

Tibial intercondylar chondroblastoma has unique clinical and imaging characteristics. Based on our experience, direct surgery rather than preoperative biopsy is suggested for treating tibial intercondylar chondroblastoma with typical clinical and imaging characteristics. Among the patients in this study, 3 patients with early lesions underwent CT-guided biopsy; the other 9 patients with confirmed clinical and imaging features did not undergo preoperative biopsy, but both intraoperative frozen pathology and postoperative pathology are suggested in chondroblastoma.

### Surgical procedures

The following aspects should be considered regarding the surgical treatment of tibial intercondylar chondroblastoma: (1) Lesions need to be completely curetted and inactivated. It was reported in the literature that the recurrence rate after curettage treatment was 3.6-35% 5. Therefore, the treatment with thorough curettage and complete inactivation should be performed for this type of tumor, which could decrease the recurrence rate as significantly as for giant cell tumors of bone. After curettage, inactivation was performed with phenol and alcohol. Relapse occurred in 1 case; after another treatment with removal and inactivation, cementation was performed. (2) Allogeneic bone grafting is recommended. Aside from the one patient who experienced relapse, the other patients recovered well after curettage and the transplantation of allogeneic bone grafts into the tumor cavity. In these cases, allogeneic bone grafting was suitable for avoiding the consequences of cutting the ilium, since the focal areas of tibial intercondylar chondroblastoma were not large or part of weight-bearing surfaces. (3) Any damage to the epiphyseal plate and penetration of the articular cavity should be carefully avoided. In these patients, no growth malformations or inequalities in the lower extremities were observed during the postoperative follow-up period. The posterior cruciate ligament insertion was fixed with suture anchors. Because the lesions were closely below the posterior cruciate ligament, the latter were all fixed with 5.0 suture anchors in these patients, and this approach was conducive to postoperative functional exercise at an early stage. Good recovery could be achieved without the postoperative application of plaster or orthosis. These patients also achieved satisfactory functional recovery of the knee joint. A posteromedial surgical approach was applied in these cases. The tibial intercondylar posterior cruciate ligament insertion was exposed when the articular capsule was incised after the medial head of the gastrocnemius was pulled toward the fibular side. In these patients, the incisions were short (5-8 cm in length), the trauma was minimal, and the operation was easy. Thus, the posteromedial approach was effective in all patients in this study.

In conclusion, tibial intercondylar chondroblastoma has unique clinical and imaging characteristics and can effectively be treated with curettage, inactivation, allogeneic bone graft transplantation and fixation with suture anchors through the posteromedial approach.

## AUTHOR CONTRIBUTIONS

Yang Z wrote the manuscript. Tao H and Ye Z contributed to the discussion. Huang X, Lin N and Yang M contributed to the discussion and provided comments on an earlier version of the manuscript. All authors have read and approved the final version of the manuscript.
